# Tree nut consumption and prevalence of carotid artery plaques: The National Heart, Lung, and Blood Institute Family Heart Study

**DOI:** 10.1007/s00394-021-02640-x

**Published:** 2021-07-19

**Authors:** Ania Stolarczyk, R. Curtis Ellison, Donna Arnett, Luc Djousse

**Affiliations:** a University of Rochester School of Medicine and Dentistry, Rochester, NY, USA; b Boston University School of Medicine, Boston, MA, USA; c College of Public Health, University of Kentucky, Lexington, KY, USA; d Brigham and Women’s Hospital and Harvard Medical School, Boston, MA, USA

**Keywords:** Epidemiology, atherosclerosis, diet, risk factors, tree nut consumption, carotid artery plaques

## Abstract

**Purpose:**

While tree nut consumption has been shown to be cardioprotective, few studies have examined the relationship between tree nut consumption and carotid atherosclerosis. We tested the hypothesis that tree nut consumption would be inversely related with carotid atherosclerosis in adults.

**Methods:**

We cross-sectionally analyzed data from 4,536 participants of the National Heart, Lung, and Blood Institute (NHLBI) Family Heart Study conducted in the United States. Dietary patterns amongst participants were variable, tree nut consumption was self-reported using the Food Frequency Questionnaire (FFQ) and B-mode ultrasound of the carotid arteries was used to assess for presence of carotid artery plaques (primary outcome) and carotid intima media thickness (cIMT). Multivariable logistic regression was used to estimate odds ratio (95% confidence interval) of prevalent carotid artery plaques and linear regression was used to estimate adjusted mean cIMT across categories of nut consumption.

**Results:**

The mean age was 52.3 years (SD=13.6), 95.6% of the participants were white, and 54% were female. The median tree nut intake was 1–3 servings/month. Odds ratios (95% CI) for prevalent carotid artery plaques were 1.0 (reference), 1.03 [0.86, 1.4], 0.89 [0.70, 1.13], and 0.96 [0.73, 1.26] for tree nut consumption of almost never, 1–3 times/month, 1/week, and 2+/week, respectively, adjusting for age, sex, race, field center, BMI, smoking status, alcohol consumption, creatinine, energy intake, fruit and vegetable consumption, exercise, and education. In secondary analysis, there was a suggestive inverse association of tree nut consumption with cIMT in the internal carotid artery, but not the common carotid or bifurcation.

**Conclusion:**

Our data showed no association between tree nut consumption and prevalence of carotid artery plaques in adults.

## 1. Introduction:

Stroke is the 2^nd^ leading cause of death worldwide [1] and is associated with higher direct and indirect costs, including residual disability in overall functioning [2]. Carotid atherosclerosis has been associated with an increased risk of stroke in both men and women over the age of 55 [3], as well as increased all-cause and cardiovascular mortality in men over age 70 [4]. Carotid intima media thickness (cIMT) is a marker of arterial injury and is a strong predictor of future cardiovascular events [5,6]. Many studies have shown that tree nut consumption is associated with several cardiovascular benefits [7–10]. Tree nuts have a high content of monounsaturated fatty acids (MUFA) and n-6 polyunsaturated fatty acids (PUFA), protein, fiber, and antioxidant vitamins like Vitamin E and selenium, which are important components of a heart-healthy diet [11–15]. Tree nut consumption has been shown to decrease blood pressure [16,17] as well as reduce total and LDL cholesterol levels [18–21]. These data suggest that tree nut consumption may favorably influence subclinical atherosclerosis, including carotid atherosclerosis [22].

Although a sub-study of the PREDIMED trial showed that supplementation of a Mediterranean diet with 30 g tree nuts/day over an average of 2.4 years resulted in a decrease in internal carotid artery IMT and plaque formation compared to the control low fat diet group [23], no previous observational studies have examined the association of tree nut consumption with carotid atherosclerosis and cIMT outside of the context of a heart healthy diet. Therefore, we examined whether tree nut consumption among participants of the National Heart, Lung, and Blood Institute (NHLBI) Family Heart Study conducted in the United States was associated with (i) prevalence of plaques in the carotid arteries and (ii) cIMT.

## 2. Materials and Methods:

### 2.1 Study population

Data were collected from participants of the NHLBI Family Heart Study, a multi-centered population study designed to assess genetic and non-genetic determinants of heart health from four US regions [the Framingham Heart Study (Framingham, MA), the Atherosclerosis Risk in Communities (ARIC) Study (sites in Forsyth County, NC and Minneapolis, MN), and the Utah Health Family Tree Study (Salt Lake City, UT)] between 1994–1995. Exclusion criteria included lack of exposure/covariate data with more than half of the questionnaires incomplete, implausible data values, as well as poor quality ultrasound. Differences in demographic characteristics were minimal in pre vs post exclusion groups. (Supplementary Table 1). Detailed description of the NHLBI Family Heart Study has been previously reported [24].

### 2.2 Assessment of tree nut consumption

Intake of tree nuts was measured using a semi-quantitative Food Frequency Questionnaire (FFQ) established by Willett et al [25] that has been validated in another study [26]. Participants were asked to indicate their average consumption of tree nuts in the past year (serving size 1 oz.), with the following pre-specified answers (servings/time): never/<once per month, 1–3/month, 1/week, 2–4/week, 5–6/week, 1/day, 2–3/day, 4–5/day, and 6+/day [27]. Due to sparse data in some categories, we collapsed adjacent categories to allow for stable estimates of effect [almost never, 1–3 servings/month, 1 serving/week, and 2+ servings/week].

### 2.3 Assessment of carotid artery plaques and intima-media thickness

Examinations to determine cIMT and presence of plaques were done using a Biosound 2000IISA carotid B-mode real-time ultrasound and were performed by trained technicians using a common scanning protocol [28]. The images were then recorded onto a VHS cassette tape and read at the central ultrasound reading center. Presence of plaques was determined by the following criteria: carotid artery wall thickness >1.5 mm, irregular shape, and abnormal wall texture as determined by differences in echogenicity [29,30]. If two of the three above criteria were met, it was determined that the participant had a plaque in a given segment and the total number of plaques per participant was recorded. Reproducibility for presence of plaques was reasonable (κ value of 0.76) [30].

Measurements of cIMT were determined in accordance with the Atherosclerosis Risk in Communities Study (ARIC) protocol [29]. cIMT was determined bilaterally on three segments: the distal common carotid artery (1 cm proximal to the dilation of the carotid bulb), the carotid artery bifurcation (1 cm segment proximal to the flow divider), and the proximal internal carotid artery (1 cm segment distal to the flow divider). Eleven adjacent areas (1 mm) were measured in the far wall of each of the six segments and the maximum value was determined for each segment [31]. Details of cIMT measurement in this study have been previously reported [28].

The reproducibility and reliability of this method of obtaining cIMT measurements and assessing presence of plaques has been previously reported [32,33].

### 2.4 Other important variables

Demographics, education, lifestyle factors, and comorbidities were self-reported using standardized questionnaires. Information on cigarette smoking was collected using a questionnaire that stratified participants into three categories: never smoker, former smoker, and current smoker. Information on alcohol use was self-reported as the number of alcoholic beverages per week on average over the course of 12 months. Height and weight were measured by study staff and used to calculate BMI. Participants were asked to fast for 12 hours and blood samples were collected to measure serum lipids and serum creatinine. The Roche COBAS FARA centrifugal analyzer (Boehringer Mannheim Diagnostics, Indianapolis) was used to measure both triglyceride concentrations using the triglyceride GB reagent as well as serum total cholesterol using a commercial cholesterol oxidase method. This method was also used to determine HDL-cholesterol concentrations after non-HDL-cholesterol precipitation with magnesium/dextran. LDL cholesterol was quantified either using ultracentrifugation on EDTA plasma or calculated by the Friedewald formula in the setting of low triglyceride levels [34].

### 2.5 Statistical analysis

Baseline characteristics were contrasted across frequencies of nut consumption. We used logistic regression to estimate odds ratios (95% CI) for prevalent carotid plaques using sequential models. After the crude model, model 2 controlled for age, sex, and race and model 3 additionally adjusted for field center, education, BMI, smoking status, alcohol consumption, exercise, energy intake, fruit and vegetable consumption, and creatinine using the lowest category of intake as reference. Previous studies have shown evidence that creatinine may be an independent risk factor for carotid artery plaques [35]. Furthermore, there has been evidence for an association of alcohol consumption and the development of atherosclerosis [36] as well as a known beneficial effect of fruit and vegetable consumption on atherosclerosis risk [37]. For secondary analysis, we fitted a multivariable linear regression model to estimate adjusted mean cIMT across frequency of nut intake with adjustment as above. Alpha level was 0.05. Covariates were selected based on a priori knowledge of risk factors for atherosclerotic disease [38].

## 3. Results:

Of the 4,536 participants included in our analyses, the mean age was 52.3 years (SD = 13.6) and 54% were female. The median tree nut consumption was 1–3 servings/month, which is slightly lower than previous US estimations [39]. Frequent consumption of tree nuts was associated with male sex, older age, higher intake of fruit and vegetables, prevalent hypertension and CHD, and higher level of physical activity (Table 1). Tree nut consumption was not associated with the prevalence of carotid artery plaques: odds ratios (95% CI) for prevalent carotid artery plaques in each category of tree nut consumption (number of servings/time) were 1.0 for almost never (reference); 1.03 [0.86, 1.4] for 1–3 servings/month; 0.89 [0.70, 1.13] for 1 serving/week; and 0.96 [0.73, 1.26] for 2+ servings/week, adjusting for age, sex, race, field center, BMI, smoking status, alcohol consumption, creatinine, energy intake, fruit and vegetable consumption, exercise, and education (Table 2).

In our secondary analysis, there was no association between tree nut consumption and cIMT for the bifurcation and the common carotid artery; however, we observed a suggested inverse relationship between tree nut consumption and internal carotid artery cIMT [adjusted mean (SE) across consecutive categories of tree nut consumption: 0.85 (0.04), 0.83 (0.05), 0.78 (0.05), and 0.79 (0.05), Table 3] but this data did not reach significance (*p* = 0.03). Categories of tree nut consumption were the same as for carotid artery plaques.

## 4. Discussion:

In this cross-sectional study, we observed that tree nut consumption was not associated with prevalent carotid artery plaques. In secondary analysis, we found suggestive evidence for an inverse association of tree nut consumption with intima media thickness of the internal carotid arteries but not for the common carotid and bifurcation. This may imply a beneficial effect of tree nut consumption on internal carotid artery plaques independent of the Mediterranean diet.

Few studies have examined the association of tree nut consumption with carotid artery plaques. A cross-sectional study of 211 patients with dyslipidemia found an inverse relationship between levels of alpha linoleic acid (ALA), found in high concentrations in tree nuts including walnuts, and both carotid and femoral artery plaque burden [40]. A recent study found that in ischemic stroke patients within seven days of onset, patients with vulnerable carotid plaques consumed fewer nuts (5.66 ± 7.1 g/d) than patients without vulnerable plaques (8.84 ± 15.9 g/d, *P*=0.024) [41]. The observed suggestive inverse relation of tree nut consumption with cIMT of the internal carotid artery in our study is concordant with the results from the PREDIMED trial [23], where a 2.4-year intervention with MedDiet supplemented with 30 g/day mixed tree nuts led to a statistically significant regression in plaque height and reduction in cIMT of the internal carotid artery when compared to the control low-fat diet. These findings are intriguing and merit further study with larger samples.

Previous studies have investigated markers of subclinical atherosclerosis. For example, in a parallel-group randomized study, patients in the lifestyle modifications (LSM) plus 80 g/day pistachios group showed statistically significant reduction in average brachial-ankle pulse-wave velocity (baPWV) and brachial artery flow-mediated vasodilation (BAFMD) after a 3 month intervention period compared to the LSM only group [42].

Furthermore, a randomized cross-over study demonstrated that subjects on a walnut diet showed improvements in endothelium-dependent vasodilation and decreased vascular cell adhesion molecule-1 after a 4-week intervention compared to the Mediterranean diet control group [43]. These findings from randomized clinical trials are supported by several potential mechanisms by which tree nuts may protect from subclinical atherosclerosis.

Are there biologic mechanisms that could make the observed inverse relationship between tree nut consumption and cIMT in the internal carotid causal? Several studies have demonstrated the beneficial effects of tree nuts on multiple levels of cardiovascular disease progression, including serum lipid profiles, blood pressure, and endothelial function. Polyphenolic flavonoids found at concentrations as high as 34 mg/100 g in tree nuts prevent ex-vivo oxidation of LDL cholesterol and thus it is plausible that tree nuts can inhibit in-vivo atherosclerotic plaque formation [44–47]. A 2015 meta-analysis of controlled intervention trials demonstrated that tree nut consumption was associated with significant decrease in LDL-cholesterol as well as in total cholesterol, ApoB, and triglycerides compared to control [22]. Furthermore, walnut consumption significantly improved flow mediated-dilation (FMD), an independent predictor of cardiovascular events, compared to control [48]. Blood pressure-lowering effects of tree nuts have been investigated in a randomized controlled feeding trial in which a diet with 18% daily energy intake derived from walnuts led to significantly reduced brachial and central mean arterial pressure compared to baseline [17]. However, other studies on tree nut consumption and markers of cardiovascular disease have yielded mixed results [49,50].

Major strengths of the present study included sampling from multiple regions across the United States, availability of several confounders including comorbidities and cardiovascular risk factors, a standardized approach to assess cIMT with excellent agreement between readers, and a standardized collection of health data including dietary intake and serum lipids for the subjects studied.

Our study has limitations. Because the present study used a cross-sectional analysis, temporality could not be inferred for the relationship between tree nut consumption and carotid plaques. Furthermore, our study population was almost exclusively white, leading to limited generalizability. The possibility of residual confounding by other factors not accounted for could have also influenced our results. In our study, tree nut consumption was self-reported, therefore subject to recall bias and we did not differentiate between types of tree nuts. In addition, we did not have information on preparation methods of tree nuts (for example, raw versus roasted with salt or honey), which may influence results as added salt and refined sugar are known to negatively affect cardiovascular health [51]. Finally, we had limited sample size in high frequency categories of nut consumption, thereby limiting our ability to detect small yet meaningful effects.

### 4.1. Conclusion

Our data are consistent with no meaningful association between tree nut consumption and prevalent carotid artery plaques. Suggested inverse association between tree nut consumption and cIMT in the internal carotid artery, but not in the common or bifurcation sites, warrants further investigation to confirm these findings and determine a mechanism by which tree nut consumption may positively influence subclinical atherosclerosis in the carotid arteries.

## Supplementary Material

1743008_Sup_Tab

## Figures and Tables

**Table 1.** Baseline characteristics according to tree nut consumption in the NHLBI Family Heart Study (N = 4536)

	Frequency of nut consumption (number of servings/time)			
	Almost never (N= 1949)	1–3/month (N= 1498)	1/week (N= 643)	2+/week (N= 446)
Age (years)	52.7 ± 13.9	50.8 ± 13.5	52.2 ± 13.3	55.8 ± 12.8
Sex, female (%)	62.1	51.7	44.3	44.0
Race, White (%)	95.4	96.5	95.8	93.5
Race, African American (%)	4.6	3.5	4.2	6.5
Field center (%)				
Framingham, MA	24.2	17.9	15.9	12.1
Salt Lake City, Utah	22.1	29.8	28.2	31.2
Minneapolis, MN	28.7	28.4	30.0	26.7
Forsyth County, NC	25.0	23.8	26.0	30.0
Anthropometric data				
BMI (kg/m^2)	27.5 ± 5.6	27.6 ± 5.3	27.8 ± 5.7	27.6 ± 5.6
Waist Girth (cm)	96.0 [86.0, 106.0]	97.0 [88.0, 106.0]	98.0 [89.0, 107.0]	98.0 [88.0, 107.0]
Nutrition and Energy Expenditure				
Energy intake (kcal)	1497 [1189, 1900]	1696 [1332, 2108]	1801 [1479, 2360]	1937 [1584, 2416]
Fruit and vegetables (servings/week)	23.0 ± 12.5	23.1 ± 11.9	25.3 ± 13.9	25.4 ± 13.1
Exercise (metminute/week)	346.2 [83.1, 858.5]	369.2 [110.8, 856.2]	369.2 [103.9, 863.1]	387.7 [136.9, 821.5]
Serum Lipids				
HDL cholesterol (mg/dl)	52 ± 16	50 ± 15	50 ± 15	50 ± 15
LDL cholesterol (mg/dl)	125 ± 36	125 ± 36	126 ± 34	127 ± 34
Triglycerides (mg/dl)	125 [84, 183]	123 [85, 184]	126 [84, 181]	130 [85, 188]
Total cholesterol (mg/dl)	205 [178, 230]	202 [177, 229]	203 [177, 227]	206 [183, 231]
Lipoprotein (a) (mg/dL)	34 [18, 59]	34 [18, 64]	37 [18, 66]	31 [18, 59]
Blood pressure				
Systolic blood pressure (mmHg)	117.7 ± 19.2	116.5 ± 17.6	118.2 ± 16.9	119.9 ± 17.8
Diastolic blood pressure (mmHg)	68.6 ± 10.3	69.4 ± 10.1	70.2 ± 9.8	70.4 ± 10.5
Kidney function				
Creatinine (mg/dL)	0.96 ± 0.30	0.96 ± 0.21	0.98 ± 0.19	1.01 ± 0.22
Smoking status (%)				
Current smoker (%)	14.9	13.2	15.9	14.6
Former smoker (%)	32.0	31.0	32.4	31.6
Never smoker (%)	53.1	55.8	51.8	53.8
Drinking status (%)				
≤7 drinks/week	41.3	38.6	39.2	36.3
>7 drinks/week	11.2	15.8	17.7	20.6
Never drinker (%)	47.6	45.7	43.1	43.1
Education (%)				
High School or less	9.4	4.9	6.4	4.7
Bachelor/Some College	39.9	37.6	37.5	39.4
Advanced Degree	50.6	57.5	56.1	55.8
Comorbidities				
Hypertension (%)	28.3	28.7	29.5	35.1
Diabetes mellitus (%)	6.4	5.9	6.2	7.9
Prevalent CHD (%)	11.1	9.8	10.9	17.0

Frequency missing – Diabetes mellitus (N=5), Hypertension (N=11)

Data reported as mean ± SD or mean [95% CI] where applicable

**Table 2.** Crude and adjusted odds ratio of prevalent carotid plaques according to tree nut consumption for participants in the NHLBI Family Heart Study

Nut consumption (1 oz serving)		Odds ratio [95% CI]		
	Number of people with plaques/N	Model 1^a^	Model 2^b^	Model 3^c^
Almost never	515/1949	1.00 (reference)	1.00 (reference)	1.00 (reference)
1–3/month	364/1498	0.89 [0.77, 1.04]	0.99 [0.83, 1.18]	1.03 [0.86, 1.24]
1/week	156/643	0.89 [0.73, 1.10]	0.85 [0.67, 1.07]	0.89 [0.70, 1.13]
2+/week	137/446	1.24 [0.99, 1.546]	0.93 [0.72, 1.20]	0.96 [0.73, 1.26]
P value for linear trend		0.45	0.28	0.51

a Crude odds ratio

b Adjusted for age, sex, and race

c Adjusted as in model 2 with additional adjustment for field center, BMI, smoking status (3 groups), alcohol consumption (3 groups), creatinine, energy intake, fruit and vegetable consumption, exercise, and education (3 groups)

**Table 3.** Crude and adjusted means (SE) for cIMT by site and tree nut intake frequency.

	Frequency of tree nut intake				
	1 (almost never)	2	3	4 (2+)	*p* trend
**Internal carotid artery**					
N	873	623	260	209	
Crude	0.82 (0.02)	0.80 (0.02)	0.79 (0.03)	0.86 (0.03)	0.86
Adjusted model^a^	0.85 (0.04)	0.83 (0.05)	0.78 (0.05)	0.79 (0.05)	0.03
					
**Common carotid artery**					
N	1622	1235	516	368	
Crude	0.74 (0.01)	0.71 (0.01)	0.74 (0.01)	0.78 (0.01)	0.10
Adjusted model	0.78 (0.01)	0.77 (0.01)	0.78 (0.01)	0.78 (0.01)	0.76
					
**Bifurcation**					
N	1380	1042	434	307	
Crude	0.98 (0.01)	0.99 (0.02)	1.00 (0.02)	1.02 (0.03)	0.20
Adjusted model	1.05 (0.03)	1.07 (0.03)	1.06 (0.03)	1.02 (0.03)	0.45

a adjusted for age, sex, race, field center, BMI, smoking status, alcohol consumption, creatinine, energy intake, fruit and vegetable consumption, exercise, and education
