# Supplementary material for: Tree nut consumption and prevalence of carotid artery plaques: The National Heart, Lung, and Blood Institute Family Heart Study
Source: Eur J Nutr. Author manuscript; Available in PMC 2023 Feb 1. (PMC8766619; doi:10.1007/s00394-021-02640-x)
Supplement: 1743008_Sup_Tab [file NIHMS1743008-supplement-1743008_Sup_Tab.docx]

| **Supplementary Table 1**: Demographic characteristics of study subjects before and after exclusions were applied. | | | | |
| --- | --- | --- | --- | --- |
|  | All subjects before exclusions | | Subjects after exclusions applied | |
|  | N | Mean (SE) | N | Mean (SE) |
| Age (years) | 5975 | 52.6 (14.1) | 4749 | 52.3 (13.7) |
| BMI (kg/m^2) | 5332 | 27.6 (5.5) | 4745 | 27.6 (5.5) |
| Waist girth (cm) | 5324 | 97.3 (15.3) | 4739 | 97.2 (15.3) |
| HDL cholesterol (mg/dL) | 5860 | 50.5 (15.3) | 4668 | 50.4 (15.2) |
| LDL cholesterol (mg/dL) | 5857 | 125.2 (35.3) | 4666 | 125.1 (35.2) |
| Triglycerides (mg/dL) | 5860 | 124.0 [83.0, 183.0] | 4668 | 124.0 [84.0, 183.0] |
| Total cholesterol (mg/dL) | 5860 | 204.0 [178.0, 229.0] | 4668 | 204.0 [177.5, 229] |
| Lipoprotein (a) (mg/dL) | 3824 | 33.0 [18.0, 62.0] | 3038 | 34.0 [18.0, 62.0] |
| Creatinine  (mg/dL) | 5870 | 0.97 (0.25) | 4675 | 0.97 (0.26) |
| Energy intake (kcal) | 5141 | 1666 [1290, 2133] | 4749 | 1659 [1299, 2098] |
| Exercise (met-min/week) | 5176 | 359 [83, 854] | 4732 | 365 [93, 858] |
| Systolic blood pressure (mmHg) | 5327 | 117.9 (18.4) | 4744 | 117.6 (18.2) |
| Diastolic blood pressure (mmHg) | 5327 | 69.3 (10.2) | 4744 | 69.3 (10.2) |
